# 

**Published:** 1867-01

**Authors:** 


### INDEX TO VOLUME VIII.

PAGE.
Association, American Medical,-- 114, 251, 331
" Military Tract, ----- 40
Ankle-Joint, A New Apparatus for. By J. S. Sherman, M.D.,- 65
Abscess, Pelvic. By M. M. Eaton, M.D.,-------------------- 71
Academy of Medicine, New York, 90
Asylum, Deaf and Dumb, of Tenn. 153
Association of Alumni, at Chicago Medical College,-----------235, 250
Alcohol, Action of. By R. L. Walston, M.D.,--------------- 258
Alcoholic Stimulants in Shock, Why do we give. By D. W. Young, M.D.,---------------- 705
Association, Fox River Med.,-284, 484
Anatomy, Act, in Pennsylvania,- 300
Address of Greeting,------------ 332
" by President, ----------- 333
" Valedictory. By R. E. McVey, M.D.,----------- 414
Announcement, Annual,-------- 442
Anæsthetic, new,---------------- 487
Alcohol in Disease. By S. Wilks, M.D.,-------------------------- 492
Alcohol, its effects on Man. By N. S. Davis, M.D.,------------ 522
Anæsthesia, local,--------------- 540
Apology, ------------------------ 626
Anatomical Specimens, --------- 696
Anatomy, Human. By T. G. Richardson, M.D., --------------- 439
Asiatic Cholera, Notes on the Origin, Nature, Prevention of. By John C. Peters, M.D.,---------- 747
Appointments, Professional,----- 748

Books not received, ------------ 117
Bread, poisoned, at Winona,----- 121
Body of Jeremy Bentham,------- 188
Botany, Indigenous. By — Massey, M.D.,-------------------- 270
Blood-letting. By C. H. Spillman, M.D.,------------------ 285
Bellevue Place,------------------ 380
Brown, Dr. Baker, of London,--- 442
Building, New College, --------- 504
Books, Medical, ---------------- 626
Bricks, Egyptian,--------------- 125
Batteries, Medical, Guide for using. By A. C. Garratt, M.D.,- 181
Belladonna in Disease of the Cornea, Action of. By Jos. S. Hildreth, M.D.,----------------- 736

Chestnut Leaves in Pertussis,---- 760

PAGE.
Coffee, Delirium Tremens successfully treated with. By Wm. B. Whitehead, M.D ,------------ 733
Close of the Volume,----------- 748
Cholera Conference, Weimar,---- 740
Cholera. By N. Wright, M.D.,-- 23
Chloroform, Death by,---------33, 378
Clinic at Mercy Hospital. By Prof. N. S. Davis,----------107, 306
College, Rush Medical, ---63, 116, 625
" Chicago Medical-------- 117, 249, 313, 625
" Harvard Medical,------ 209
Convention, Medical, in Wisconsin 504
Cholera and Quarantine,--------- 123
Congress, International,-------- 123, 662, 665, 694
Carbuncle, Treatment of By T. L. Leavitt, M.D.,------------- 172
Cancer, Remarks on. By J. O'-Reilly, -------------- ----- 175
Convention of Delegates from Medical Colleges,-------------245, 353
Commencement, Chicago Medical College,---------------------- 249
Cholera, Pathology of. By W. Duff Greene, M.D.,----------- 286
Corneal Anæsthesia. By Jos. S. Hildreth, M.D.,------------ 290
Cod-Liver Oil. By A. Bissell, M.D. 295
Clinic, Charitable. By J. S. Hildreth, M.D., -------------314, 626
Curvature, Spinal. By J. S. Sherman, M.D.,----------------- 328
Cholera Papers,----------------- 349
Constitution, Amendments to,--- 349
Compensation to the Secretary,-- 349
Committees, Standing and Special, 425
Committees,--------------------- 432
Chloroform, Dangers from, ------ 487
Cerebro-Spinal Meningitis. By F. R. Payne, M.D.,-------------- 513
Chloroform, Ratio of Deaths in England, -------------------- 538
Cervix Uteri, Excision of,------- 546
Chicago, Medical Education in,-- 560
Corneæ. Panniform. By J. S. Hildreth, M.D.,----------------- 577
Cholera in 1866. By H. Wardner, M.D.,------------------ 582
Chloroform in Fevers. By Geo. F. Brickett, M.D.,------------ 594
Colleges, Medical,-------------- 627
Chloroform, Death from,-------- 697
Cholera in Chicago. By Prof. N. S. Davis,----------------- 641

PAGE.
Children, Bowel-Affections of. By Prof. N. S. Davis, ---------- 672
Cholera, its Origin, Nature, etc. By J. C. Peters, M.D., -------- 439
Cholera, Report on Epidemic, --- 557
Chemistry. By Wm. T. Brande, - 622
Diseases of Women, Lectures on. By Charles West, M.D., ------- 748
Drunkenness, Head-quarters of, -- 121
Delegates to the International Congress, 184
Development, Suspended, in Twin Pregnancies. By M. M. Eaton, M.D., ---------------- 210
Delegates to American Medical Association, ---------------- 434
Death from Pyæmia, ----------- 437
Digestion, Observations on. By R. Dexter, M.D., ------------ 472
Diabetes, --------------548, 550, 552
Disinfection, Principles and Practice of. By R. Bartholomew, M.D., ---------------------- 556
Diphtheria. By E. S. Gaillard, M.D., ---------------------- 111
Diseases and their Treatment, Index of. By T. H. Tanner, M.D. 53
Dissections, Practical. By R. M. Hodges, M.D., ------------- 301
Duffield, Park, & Co., ----------- 557
Ear Disease, Clinical Papers on. By D. B. St. John Roosa, M.D., 730
Epidemics, A Paper on. By H. Nance, M.D., -------------- 5
Essay on Cholera. By N. Wright, M.D., ---------------------- 23
Elephantiasis. By R. Dexter, M.D. 35
Essay. By N. Holton, M.D., --- 76
Electricity, Medical Use of. By Drs. Beard & Rockwell, -98, 225, 692
Exposition at Paris, ------------ 123
Evil, Social, ------------------ 140
Entertainments and Receptions, - 333
Essays, Prize, ---------------- 340
Entertainment at Hon. Geo. H. Pendleton's, ---------------- 343
Excursion, Steamboat, ---------- 347
Election of Members, ----------- 419
Education, Medical, ------------ 440
Erysipelas treated by Sulphite of Soda, ---------------------- 632
Epilepsy, Curare in, ------------ 636
Epidemics, Notes on. By F. E. Anstie, M.D., -------------- 113
Epidemics, Common Nature of. By S. Smith, M.D., ---------- 114
Ethics, Medical, Code of, -------- 302

PAGE.
Forceps, A new Ecraseur. By E. Andrews, M.D., -------------- 722
Fever, Breakbone, History of. By R. Fraser Michel, M.D., ------- 212
Fever, Typhoid, Use of Chloride Sodium, -------------------- 233
Feet, Deformities of. By J. S. Sherman, M.D., ------------- 465
Fœtus, Suspension of Growth. By S. D. Mercer, M.D., --------- 597
Fracture of Lower Jaw, Treatment 631
Gleet, Insufflation of Medicated Powders, ------------------ 125
Gazette, Medical, -------------- 695
Glucosuria, Citrate of Soda in, --- 697
Headaches: their Causes and Cure. By Henry G. Wright, M.D., --- 747
Half-Yearly Compendium of Medical Science, ---------------- 757
Human Life, Average Duration of, 123
Hydrophobia. By A. Sawyer, M.D. 282
" By D. T. Nelson, -- 321
Hemorrhage and Abortion, Case of. By D. B. Trimble, M.D., ------ 410
Hospitals of Liverpool, --------- 487
Hospital, Cook County, --------- 561
Health, Public, ---------------- 626
Heat as a Resuscitating Agent, -- 697
Injections, a New Instrument for, 122
Intestine Wound. By Drs. Mason & Whitney, ---------------- 21
Items, Clinical. By T. A. Bennet, M.D., -------------------- 113
Insane, Injustice to, ------------ 184
Instruction, Summer Course of, -- 185
Insane, Chronic, --------------- 352
Injection, Hypodermic. By S. P. Duffield, Ph. D., ------------- 373
Insurance, Homœopathic Life. By S. R. Millard, M.D., 526
Instruction, Clinical, at Chicago Eye and Ear Infirmary, ------ 567
Inverted Uterus. By T. A. Emmet, M.D., ------------------- 113
Injuries of Eye, etc. By George Lawson, M.D., --------------- 624
Inhalations in Diseases of Respiratory Passages, ------------- 302
Indigestion. By T. K. Chambers, M.D., -------------------- 303
Inhalation: its Therapeutics and Practice. By J. S. Cohen, M.D. 747
Ill. State Medical Society, Transactions of ------------------- 748
Is it I? By H. R. Storer, M.D., - 692
Journal, Chicago Medical, ------- 315

PAGE.

Journal, Quarterly, of Medical Jurisprudence, etc., ----------441, 557
Journals, New Medical,---------- 560
Journal, American, of the Medical Sciences,-------------------- 695
Knee-Joint, Inflammation of. By J. S. Sherman, M.D., --------- 1
Lecture. By J. Brown, M.D.,--- 129
" By Dr. H. R. Storer, -- 314
Literature, Medical,------------ 343
London,---------------------- 487
Laryngoscopy. By H. A. Johnnon, M.D., ----------------- 672
Locomotor Ataxia,-------------- 687
Lecture Season,----------------- 694
L'Evenement Medical, --------- 695
Lessons on Surgical Diseases. By Prof. Velpeau, --------------- 114
Life, Renewal of. By T. K. Chambers, M.D., 179
Lexicon, Medical, for Vest Pocket. By D. B. St. J. Roosa, M.D.,-- 181
Medicine, Surgery, and Allied Sciences, Biennial Retrospect of. By H. Power and others.------ 747
Meningitis, Epidemic, or Cerebro-Spinal Meningitis. By Alfred Stillé, M.D.,----------------- 747
Medicine and Surgery applied to the Diseases and Accidents incident to Women, The Practice of. By Wm. H. Byford, M.D.,---- 746
Mortality for December, --------- 64
" January, --------- 120
" February, -------- 253
" March, ---------- 316
" April, ----------- 381
" May, ----------- 443
" June, ----------- 504
" July, ----------- 568
" August, ---------- 634
" September, -------- 699
" October, --------- 759
Motion, Retrogressive, in Birds. By S. W. Mitchell, M.D.,------ 154
Milk consumed in New York and vicinity, --------------------- 209
Mastitis and Treatment. By R. S. Addison, M.D., ------------ 279
Medicines, Unofficinal, --------- 350
Medicine, Practical, Report on. By T. L. Hewins, M.D., ------ 449
Metritis, Chronic, -------------- 545
Medicine, Science and Practice of. By W. Aitken, M.D.,--------52, 244
Medicine, Principles and Practice of,-------------------53, 556, 624

PAGE.

Marriages, Consanguineous, ----- 566
Milk, Liebig's Artificial, ------- 696
Medicine, Action of. By F. W. Headland, M.D.,------------- 242
Notice, ---------------------- 748
Notes upon General Science,----- 118
Nerve Action, Rapidity of,------ 126
Nominations and Places of Meeting,------------------344, 348
Nominating Committee,--------- 420
Nurses, School for, ------------ 487
Nervous System, Injuries of. By J. E. Erichsen, M.D.,--------- 499
Nervous Disorders, Functional. By C. H. Jones, M.D., 113
Ovariotomy. By D. Mason, M.D., 1[illegible]
Opium-Eater, cure of. By E. Andrews, M.D., --------------- 67
Onions in Intermittent Fever, --- 152
Obstetrics, Report on. By H. Nance, M.D., 392
Officers, --------------------- 421
Obituary,-----209, 441, 502, 561, 565
Oil, Olive, for Gall-Stones. By Ira Hatch, M.D., ------------ 469
Organization, Medical, in Canada, 563
Ozone, Density of, ------------ 700
Organs, Reproductive, in Childhood. By W. Acton, M.D., -- 180
Orthopedics, By D. Prince, 182, 242
Obstetrics. By C. D. Meigs, M.D. 301
Pathology and Therapeutics, Studies in. By Sam'l Henry Dickson, M.D.,------------------ 747
Prolonging Life, Hufeland's Art of. By Erasmus Williams, M.D. 747
Promotion. Dr. Conneau,-------- 64
Pocket Record, Physician's,------ 117
Paste, White,------------------ 123
Poisoning by Strychnine. By J. S. Sherman, M.D.,------------ 138
Plates, Anatomical, Method for Producing,----------------- 188
Pharmacy, Chicago College of,--- 240
Population of City and Country in England,------------------ 317
Papers presented,--------------- 341
Physicians, Female,----------343, 351
Personal, --------------------- 380
Potassa, Chlorate, in Pneumonia,- 506
Poisoning by Strychnine. By S. Hemenway, M.D.,------------ 528
Phthisis, Diabetic, and Treatment, 541
Physicians,--------------569, 636, 701
Phthisis in Barbadoes,---------- 635
Pills, Phosphorus,-------------- 697
Physician's Visiting List,------- 624

PAGE.
Prostitution in Europe. By E. Andrews, M.D., 603
Prizes offered by Connecticut Medical Society, 630
Pulse Tracings. By H. A. Johnson, M.D., 659
Prostitution and Syphilis, 693
Physiology and Pathology of the Mind. By H. Maudsley, M.D., 554
Physiology, Human, 623, 691
Proceedings of the Am. Pharmaceutical Association, 111
Paralysis, Infantile. By C. F. Taylor, M.D., 113

Report on Sanitary Condition of Chicago. By N. S. Davis, M.D. 749
Report. By Prof. N. S. Davis, 80
Receipts, Money, 189, 254, 315, 380, 443, 506, 636, 701, 758
Report on Medicine. By S. J. Young, M.D., 193
Rheumatism, Treatment of, 316, 379
Report on Sanitary Condition of City. By N. S. Davis, M.D., 329
Receptions, private, 333
Report of Medical Men in Navy, 335
Report on Insanity, 344
Resolution of Thanks, 349
Remedies, Concentrated Organic. By F. R. Payne, M.D., 385
Report of Committee to urge the Enactment of Suitable Laws by State Legislature, 428
Report of Committee on Publication, 429
Remarks, Clinical, 437
Report on Prevalence of Disease. By N. S. Davis, M.D., 475
Register, Medical. By J. M. Toner, M.D., 245
Relations of Electricity to Causes of Disease, 112

Social Evils, on the, with a Plan for their Diminution, and a Plea for the Innocent and Helpless. By Geo. J. Zeigler, M.D., 724
Sympathetic System of Nerves. By W. Murray, M.D., 623
Surgery, Aural, 103
Saffron, Spanish. By H. Biroth, 325
Spectacles, Metallic, 120
Solvent, A Remarkable, 122
Sections, Meeting of the, 332
Statistics, Medical, in the Army, 342
Spine, Trephining of, 437
Sea-Sickness, Treatment of, 487
Surgeons, English Country, 487
Surgeon J. M. Baxter, U.S. Vols. 506

PAGE.
Society, Ill. State Medical, 116, 314, 418, 440
" Chicago " 36, 54, 185, 313, 500, 558, 626
" Quincy " 48, 535
" Adams Co. " 530
" Morgan Co. " 43, 186, 363, 478, 681, 684
" Pulaski Co. " 283
" Ind. State " 315
" Clark Co. " 482
" Jackson Co. " 486
Society, New Sydenham, 564
Surgery, Contributions to. By J. Mettauer, M.D., 616
Students, French, 633
Sloughing produced by local Anæsthesia, 635
Statistics of Cholera in Italy, 636
Surgery, Ophthalmic. By J. Q. Lawrence, M.D., 53
Surgery, Uterine. By J. Marion Sims, M.D., 53
Surgery, Conservative. By H. G. Davis, M.D. 53
Statistics, Prize Essay on. By F. B. Hough, M.D., 556
Spine, Angular Curvature of. By B. Lee, M.D., 243
Spine, Injuries of. By J. Ashhurst, A.M., M.D., 302

Tetrachloride of Carbon as an Anæsthetic, Trial of—Dangerous Effects. By E. Andrews, M.D., 720
Tracheotomy. By E. Andrews, M.D., 67
Tube, Eustachian, 124
Tree, Cinchona, 348
Tape Worm. By S. A. McWilliams, M.D., 474
Transactions of American Medical Association, 693
Therapeutics, Practical. By E. J. Waring, M.D., 54

University of Michigan, Medical Department, 61
Urethra, Interior of. By E. Andrews, M.D., 211
Urine, Incontinence of, 554

Vis Conservatrix. By F. R. Payne, M.D., 589

Wound of Intestine. By Drs. Mason & Whitney, 21
Wanted, 442
Watson Abridged. By Thomas Watson, M.D., 243
Winter Retreat for Consumptives, 760

### BLISS & SHARP,

Wholesale & Retail

### DRUGGISTS AND CHEMISTS,

**144 LAKE STREET, CHICAGO,**

Keep constantly on hand a large assortment of

**PURE DRUGS FINE CHEMICAL,**

THAYER'S FLUID EXTRACTS & SUGAR-COATED PILLS,

**Keith's Preparations,**

Agents for the sale of

**Tiemann's Celebrated Surgical Instruments,**

Trusses, Elastic Stockings, Bandages, &c.

**Particular Attention Paid to Physicians' Orders.**

---

THE

### CHICAGO MEDICAL EXAMINER.

**N. S. DAVIS, M.D., EDITOR.**

A MONTHLY JOURNAL

DEVOTED TO THE

EDUCATIONAL, SCIENTIFIC, AND PRACTICAL INTERESTS

OF THE

MEDICAL PROFESSION.

The EXAMINER will be issued during the first week of each month, commencing with January, 1860. Each number will contain 64 pages of reading matter, the greater part of which will be filled with such contents as will directly aid the practitioner in the daily practical duties of his profession.

To secure this object fully, we shall give, in each number in addition to ordinary original articles, and selections on practical subjects, a faithful report of many of the more interesting cases presented at the Hospitals and College Cliniques. While aiming, however, to make the EXAMINER eminently practical, we shall not neglect either scientific, social, or educational interests, of the profession. It will not be the special organ of any one institution, society, or clique; but its columns will be open for well-written articles from any respectable member of the profession, on all topics legitimately within the domain of medical literature, science, and education.

Terms, $3.00 per annum, invariably in advance.

